# New Anti‐Angiogenic Therapy for Glioblastoma With the Anti‐Depressant Sertraline

**DOI:** 10.1002/cam4.70288

**Published:** 2024-10-23

**Authors:** Nobushige Tsuboi, Yoshihiro Otani, Atsuhito Uneda, Joji Ishida, Yasuki Suruga, Yuji Matsumoto, Atsushi Fujimura, Kentaro Fujii, Hideki Matsui, Kazuhiko Kurozumi, Isao Date, Hiroyuki Michiue

**Affiliations:** ^1^ Department of Neurological Surgery Okayama University Graduate School of Medicine, Dentistry, and Pharmaceutical Sciences Okayama Japan; ^2^ Neutron Therapy Research Center Okayama University Okayama Japan; ^3^ Department of Physiology Okayama University Graduate School of Medicine, Dentistry and Pharmaceutical Sciences Okayama Japan; ^4^ Department of Neurosurgery Hamamatsu University School of Medicine Shizuoka Japan

**Keywords:** anti‐angiogenic therapy, antidepressant sertraline, drug repositioning, glioblastoma, tumor derived endothelial cells

## Abstract

**Background and Aims:**

Anti‐angiogenic therapies prolong patient survival in some malignancies but not glioblastoma. We focused on the relationship between the differentiation of glioma stem like cells (GSCs) into tumor derived endothelial cells (TDECs) and, anti‐angiogenic therapy resistance. Especially we aimed to elucidate the mechanisms of drug resistance of TDECs to anti‐angiogenic inhibitors and identify novel anti‐angiogenic drugs with clinical applications.

**Results:**

The mouse GSCs, 005, were differentiated into TDECs under hypoxic conditions, and TDECs had endothelial cell characteristics independent of the vascular endothelial growth factor (VEGF) pathway. In vivo, inhibition of the VEGF pathway had no anti‐tumor effect and increased the percentage of TDECs in the 005 mouse model. Novel anti‐angiogenic drugs for glioblastoma were evaluated using a tube formation assay and a drug repositioning strategy with existing blood–brain barrier permeable drugs. Drug screening revealed that the antidepressant sertraline inhibited tube formation of TDECs. Sertraline was administered to differentiated TDECs in vitro and 005 mouse models in vivo to evaluate genetic changes by RNA‐Seq and tumor regression effects by immunohistochemistry and MRI. Sertraline reduced Lama4 and Ang2 expressions of TDEC, which play an important role in non‐VEGF‐mediated angiogenesis in tumors. The combination of a VEGF receptor inhibitor axitinib, and sertraline improved survival and reduced tumor growth in the 005 mouse model.

**Conclusion:**

Collectively, our findings showed the diversity of tumor vascular endothelial cells across VEGF and non‐VEGF pathways led to anti‐angiogenic resistance. The combination of axitinib and sertraline can represent an effective anti‐angiogenic therapy for glioblastoma with safe, low cost, and fast availability.

## Graphical abstract

Mouse glioma stem like cells‐derived endothelial cells compensate for the reduction of tumor blood vessels by anti‐angiogenic therapy in VEGF pathway. The combination of anti‐depressant sertraline and VEGF inhibitor is a novel anti‐angiogenic strategy for glioblastoma.

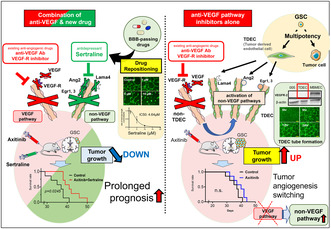

## 1 Introduction

Glioblastoma is a lethal malignant brain tumor that is characterized by a high degree of angiogenesis and tumor invasion [1]. Even with new advances in multidisciplinary treatments for glioblastoma, the median survival is still < 2 years [2, 3, 4]. Most anti‐cancer drugs, including molecularly targeted drugs, are ineffective for glioblastoma owing to the presence of the blood–brain barrier (BBB) and various other brain‐specific obstacles [5, 6].

Anti‐angiogenic therapy for malignant tumors aims to suppress tumor growth by inhibiting tumor vascular growth, thus cutting off nutrients and oxygen to the tumor [7, 8]. Bevacizumab, a humanized monoclonal antibody to vascular endothelial growth factor A (VEGFA) and an anti‐angiogenic treatment, is an effective treatment for several malignant tumors including colorectal, ovarian, and non‐small cell lung cancer [9, 10, 11]. Recent phase III clinical studies of three VEGF inhibitors (VEGF antibody, VEGFR‐2 antibody, and VEGFR‐TK inhibitor) for colorectal cancer have reported efficacy [12, 13, 14]. Bevacizumab has been approved by the FDA for recurrent glioblastoma since it prolongs progression free survival and reduces steroid requirement [15]. However, bevacizumab did not demonstrate therapeutic effects in glioblastoma in randomized phase III trials (AVAglio, RTOG‐0825, EORTC‐26101) [16, 17]. Furthermore, there are various reports on the formation of tumor blood vessels, including angiogenesis, and vascular mimicry, with different constitutive vascular endothelial cells and expressed genes [18].

Tumor derived endothelial cells (TDECs) are derived from glioma stem like cells (GSCs) [19]. GSCs differentiate into ectodermal cells and vascular endothelial cells, which are mesodermal [20, 21]. However, there are few reports on the resistance to treatment with TDECs in glioblastoma, and there are no reports of TDECs as therapeutic targets.

In this paper, we examined the mechanism of resistance by TDECs to anti‐angiogenic therapy targeting the VEGF pathway and developed a novel anti‐angiogenic drug for clinical application. Our results identify sertraline as a potential new anti‐angiogenic therapy targeting TDECs.

## 2 Materials and Methods

### 2.1 Cell Culture

The mouse GSC, 005, was provided by Dr. Tomotoshi Marumoto and Dr. Inder Verma and cultured as previously described [22]. To differentiate into TDECs, 005 cells were cultured in the EGM‐2 BulletKit medium (Lonza, Basel, Switzerland) under hypoxic conditions (1% O_2_). U87ΔEGFR was provided from Dr. Balveen Kaur (University of Texas Health Science Center, Houston, TX, USA) and cultured in Dulbecco's Modified Eagle's Medium (DMEM) with 10% fetal bovine serum (37°C, 5% CO_2_). We cultured human umbilical vein endothelial cells (HUVECs) (Takara Bio Inc., Shiga, Japan) in EGM‐2 BulletKit medium, mouse brain microvascular endothelial cells (MBMECs) (Cell Biologics, Chicago, USA) in a Complete Medium Kit With Serum and culture boost‐R (4Z0‐500‐R, Cell Systems, Kirkland, USA).

### 2.2 Animals and Animal Model

All animal studies were performed following Okayama University ethical guidelines for experimental animal care (OKU‐2018838, OKU‐2019568, OKU‐2020793, OKU‐2021590). 6‐week‐old female BALB/c‐nu/nu mice were purchased from SHIMIZU Laboratory Supplies Co, Ltd. (Kyoto, Japan). 005 (5 × 10^4^ cells) or U87ΔEGFR (3 × 10^5^ cells) were transplanted into the right frontal lobe of generally anesthetized mice. We administered LEAF (Ultra‐LEAF Purified anti‐mouse VEGF‐A Antibody, BioLegend, San Diego, USA: 2.5 mg/kg, twice a week) or Axitinib (S1005, Selleck, Houston, USA: 25 mg/kg, dissolved in Polysorbate 80 and acidified water, every other day) or vehicle solution intraperitoneally, starting on Day 14 (005) or Day 5 (U87ΔEGFR) after tumor cell transplantation. Sertraline hydrochloride (S0507, Selleck: 25 mg/kg) or diluted water was injected subcutaneously daily starting on Day 14 for the 005 mouse model.

### 2.3 Gene Analysis

Total RNA was isolated from 005, TDECs differentiated by the method described above, TDECs treated with sertraline (5 μM) and MBMECs using RNeasy kit (Qiagen, Santa Clarita, CA, USA), and samples were analyzed using Poly(A) mRNA Magnetic Isolation Module (New England Biolabs, MA, USA) for Poly(A) RNA preparation, NEBNext UltraII Directional RNA Library Prep Kit for Illumina (New England Biolabs, MA, USA) for library preparation and NovaSeq 6000 (Illumina Inc., SanDiego, CA) for sequencing. The RNA sequence analyses were performed by Rhelixa (Tokyo, Japan).

A significant change in gene expression was defined as an absolute fold change in expression of 2.0 with a *p* < 0.05 or a *q* < 0.05 compared with appropriate controls. The RNA sequence data were deposited in the Gene Expression Omnibus (GEO) under accession number GSE199495 (https://www.ncbi.nlm.nih.gov/geo/query/acc.cgi?acc=GSE199495). The data were analyzed with GraphPad Prism9 (GraphPad, San Diego, CA, USA) and RIAs provided by Rhelixa. Enrichment analysis was performed through Metascape (https://metascape.org/gp/index.html#/main/step1).

### 2.4 Tube Formation Assay

TDECs (3 × 10^4^ cells/well) and MBMECs (3 × 10^4^ cells/well) were seeded on Matrigel Growth Factor Reduced Basement Membrane (354230, Corning). Cells were treated with anti‐VEGF antibodies and various drugs. After 12 or 24 h, images were obtained, and the total tube length was measured with fluorescence microscope (BZ‐X800; Keyence).

### 2.5 Cell Proliferation Assay

TDECs (2 × 10^4^ cells/well), U87ΔEGFR (1 × 10^4^ cells/well), and MBMECs (1 × 10^4^ cells/well) were seeded in 96‐well plates. After 24 h, cells were treated with drugs. Cells were then incubated with WST‐1 and absorbance at 438 nm was measured at 30 min, 1 h, 2 h, and 4 h.

### 2.6 Drug Screening

Nineteen drugs were adjusted to final concentrations of 0, 1, 5, and 10 μM, and tube formation assay and cell proliferation assay were performed. The IC50 was calculated using Excel software.

### 2.7 Magnetic Resonance Imaging (MRI)

MRI was taken with the approval of the animal study protocol (OKU‐2021590). Glioma model mice were generally anesthetized with isoflurane and given a gadolinium contrast medium (Gadovist, Bayer, Japan). The mice were screened with high‐resolution axial T2‐weighted images using a Rapid Acquisition with Relaxation Enhancement (RARE) sequence to evaluate brain tumor size and to monitor its evolution stage, using repetition time (TR)/effective echo time (TE) = 1200/8 ms. MRI data of the mice were acquired and processed by using ParaVision 5.1 software (Bruker BioSpec 4.7 T, Ettlingen, Germany).

### 2.8 Immunohistochemistry (IHC)

Samples were treated with primary antibody (CD31: DIA‐310, Dianova, LAMA4: PAB26919, Abnova) and secondary antibody (anti‐Rat antibody: A11007, Invitrogen, anti‐Rabbit antibody: ab150072, Abnova). The samples were observed by confocal laser microscope ZEN (ZEISS, Germany). To avoid differences due to the choice of field of view, all tumors were observed at weak magnification so that all blood vessels within the tumor in one slide could be counted. To avoid subjective judgments, observations, measurements, and evaluations were performed by two or more skilled experimenters; luminal structures that expressed CD31 and had no tumor cells or other cells inside were counted as vessels.

### 2.9 Western Blotting

Proteins from 005 cells cultured in various conditions were separated by SDS‐PAGE and transferred to a PVDF membrane. The membrane was probed with rabbit anti‐VEGF Receptor 2 antibody (Cell Signaling Technology) and mouse anti‐β‐actin antibody (A5441, Sigma‐Aldrich), followed by probing with HRP‐labeled anti‐rabbit IgG (#7074S, Cell Signaling Technology) and HRP‐labeled anti‐mouse IgG (#7074S, Cell Signaling Technology). Bands were detected using an ECL Prime kit (GE Healthcare).

### 2.10 Statistical Analysis

All data are represented as the mean standard error of the mean (SEM). Kaplan–Meier survival curves were generated by using GraphPad Prism9 software and the log‐rank (Mantel‐Cox) test was performed to assess statistical significance between groups. The Unpaired Student's t‐test was used to compare two groups. Comparisons between multiple groups were performed with one‐way ANOVA with Tukey's multiple comparisons tests. *p*‐values were designated as *p < 0.05, **p < 0.01, ***p < 0.001, ****p < 0.0001, and ns non‐significant (*p* ≧ 0.05).

## 3 Results

### 3.1 Differentiation of TDECs From Pluripotent 005 GSCs

**FIGURE 1:**
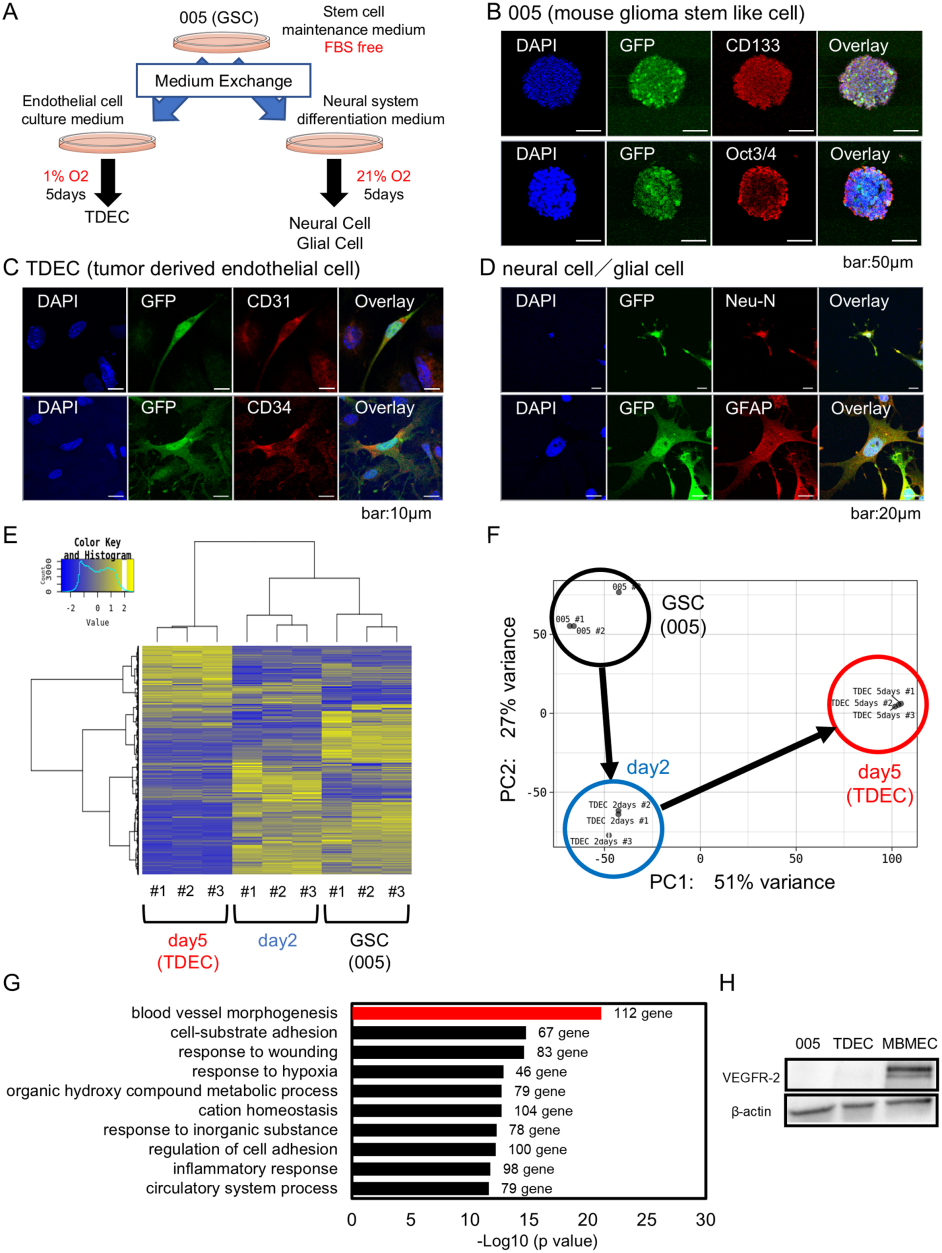
Tumor‐derived endothelial cells (TDECs) differentiated from pluripotent glioma stem like cells (GSCs, 005). (A) Schema of differentiation of 005, a mouse GSC line, to TDECs, neural, and glial cells. (B–D) Immunocytochemistry of 005 cells (B) and TDECs (C), neural cells, and glial cells (D) differentiated from 005 cells. (E, F) Heatmap and principal component analysis (PCA) of genes of 005 cells, TDECs on Day 2, and TDECs on Day 5 detected by RNA‐Seq (*n* = 3). (G) Enrichment analysis of GO terms associated with up‐regulated genes in TDECs compared with genes in 005 cells. (*q* < 0.05, log_2_ fold change (gene expression of TDECs/005 s) > 1). (H) Western blot analysis of VEGFR‐2 in 005 cells, TDECs, and mouse brain microvascular endothelial cells (MBMECs).

We differentiated 005 cells with GFP into TDECs or neural/glial cells (Figure 1A) or checked stem cell markers such as Oct3/4 and CD133 [22] (Figure 1B). After 5 days of culture in specific conditions, the differentiated cells were confirmed as TDECs or neural and glial cells, as shown by endothelial cell markers CD31 and CD34 or Neu‐N and GFAP, respectively (Figure 1C,D, and Figure S1A). Nevertheless, U87ΔEGFR did not differentiate into endothelial cell in same way (Figure S1B). The RNA‐seq in 005 and TDECs on Days 2 and 5 of differentiation was performed to evaluate the process of differentiation GSCs to TDECs. Hierarchical clustering heatmap and principal component analysis (PCA) showed that each group differentiated over time (Figure 1E,F). Enrichment analysis was then performed between 005 and TDECs on Day 5, and the top gene signature was blood vessel morphogenesis (112 genes) (*q* < 0.05, fold change (gene expression of TDEC/005) > 2, −LogP = 21.15205) (Figure 1G and Figure S2). Western blot analysis showed that VEGFR‐2 was not expressed in TDECs (Figure 1H).

### 3.2 Effect of VEGF Pathway Inhibitors (Anti‐VEGF Antibody or VEGFR Inhibitor) on Brain Tumor Model Using Two Types of Cell Lines

**FIGURE 2:**
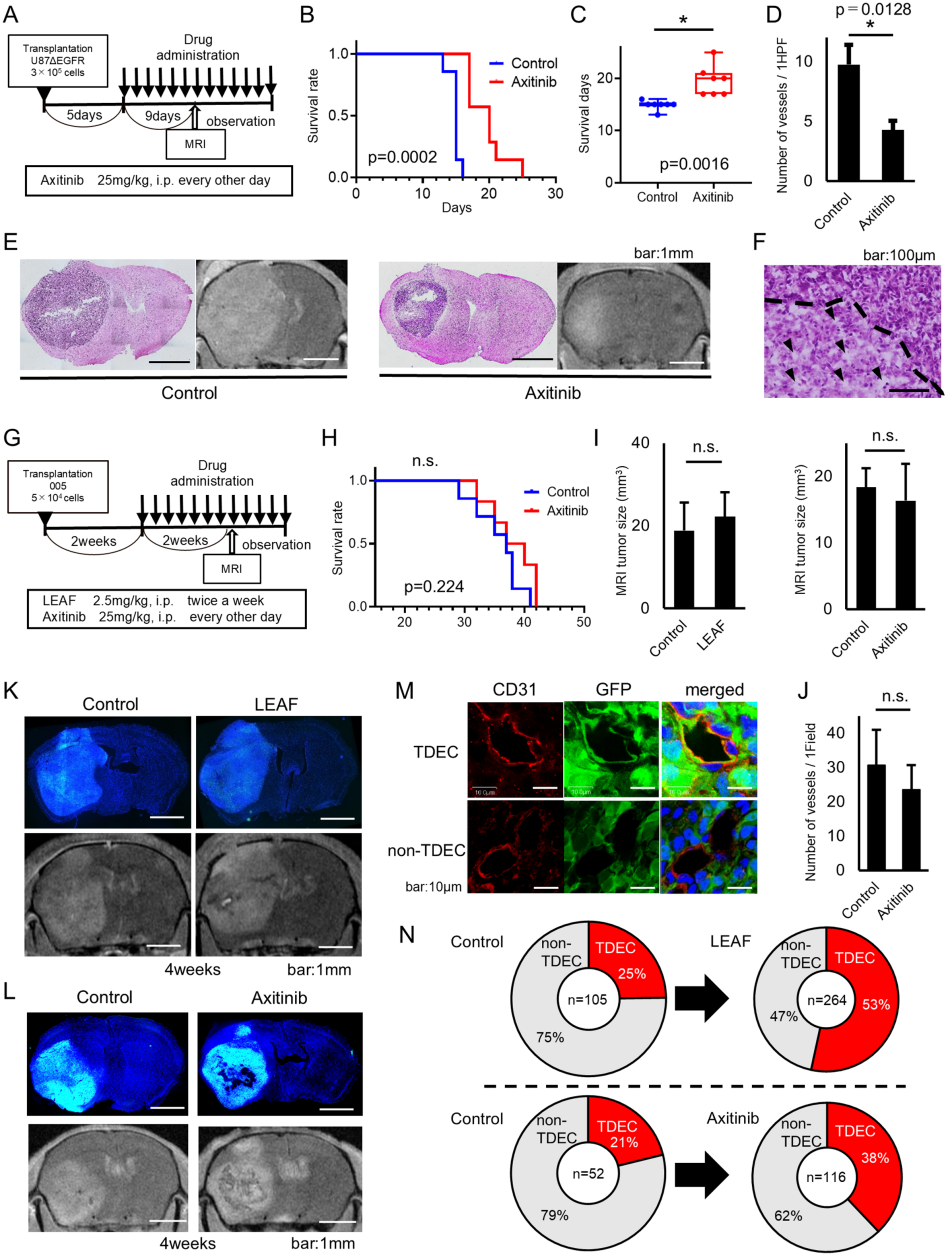
Inhibition of the VEGF pathway is ineffective in the GSC‐transplanted mouse tumor model and results in tumor vascular growth composed of TDECs. (A) Administration protocol for axitinib (VEGFR inhibitor) to mice transplanted with U87ΔEGFR cells. (B) Kaplan–Meier survival curve of control and axitinib‐treated U87ΔEGFR brain tumor model mice (log‐rank test, *p* = 0.0002). (C) Box plot for survival (days) of the control and the axitinib groups (unpaired *t*‐test, *p* = 0.0016). (D) Graph of the average number of blood vessels per field of view in tissue immunostaining in the control and the axitinib groups. (E) HE staining and gadolinium contrast‐enhanced MRI of two groups on Day 9 after the start of treatment (bar: 1 mm). (F) Intra‐tumor necrosis image (HE staining) in the axitinib group (below the dotted line: Necrotic area; arrowhead: Necrotic cells). (G) Administration protocol for axitinib and LEAF (anti‐mouse VEGF antibody) to brain tumor mouse model derived from 005 cells. (H) Kaplan–Meier survival curve of control and axitinib‐treated 005 mouse model (log‐rank test, *p* = 0.224). (I) Comparison of tumor volume on MRI (control vs. LEAF: *p* = 0.7266; control vs. Axitinib: *p* = 0.7606). (J) Comparison of the average number of blood vessels per field of view in tissue immunostaining (control vs. axitinib: *p* > 0.05). (K, L) Nuclear staining of tissue and gadolinium contrast‐enhanced MRI 2 weeks after initiation of treatment (bar: 1 mm). (M) Comparison of TDECs and non‐TDECs of intra‐tumor vascular endothelial cells by immunohistochemistry of CD31 (CD31: Red, 005: Green by GFP, nuclear staining: Blue). (N) Number of tumor blood vessels in the tumor after anti‐angiogenic therapy (LEAF, axitinib) and the ratio of TDEC/non‐TDEC in immunohistochemistry. * indicates significant differnce.

Next, U87ΔEGFR brain tumor model mice were treated with axitinib, VEGFR and multi‐kinase inhibitor (Figure 2A). Median survival time was 15 days in the control group and 20 days in the axitinib group (log‐rank test, *p* = 0.0002, Figure 2B). Average survival time was 14.9 ± 0.32 days in the control group and 19.6 ± 1.03 days in the axitinib group (unpaired *t*‐test, *p* = 0.0016, Figure 2C). CD31 immunohistochemical staining revealed the number of tumor blood vessels (HPF, 10×) in the axitinib group (4.27 ± 0.78/1HPF) and in the control group (9.75 ± 1.65/1HPF) (Unpaired *t*‐test, *p* = 0.0128, Figure 2D). Furthermore, HE staining and MRI results of tumors 9 days after drug administration demonstrated a remarkable tumor suppressive effect and necrotic lesion in the axitinib group (Figure 2E,F).

The 005 model with axitinib showed 37 days (control) and 38.5 days (axitinib) in median survival time, respectively (log‐rank test, *p* = 0.224, Figure 2G,H). The tumor volume (mean ± SEM) on MRI at 2 weeks after drug administration was 18.85 ± 6.82 mm^3^ (control) and 22.25 ± 5.90 mm^3^ (LEAF) (unpaired *t*‐test, *p* = 0.727); the tumor volumes were 18.35 ± 2.85 mm^3^ (control) and 16.34 ± 5.59 mm^3^ (axitinib), respectively (Unpaired *t*‐test, *p* = 0.761) (Figure 2I–L). Furthermore, the number of tumor blood vessels was 30.7 ± 10.4/1HPF (control) and 23.6 ± 7.2/1HPF (axitinib) (Unpaired *t*‐test, *p* = 0.3920, Figure 2J). Together these results indicate that LEAF and axitinib treatment had no tumor reduction effect on tumors derived from 005.

TDECs or non‐TDECs, which were endothelial cells derived from normal brain tissue, bone marrow, or mesenchymal stem cells, but not differentiated from GSCs, were evaluated by the CD31immunostaining and 005‐derived GFP (Figure 2M). In LEAF group and axitinib group, both the proportion and number of non‐TDECs decreased compared with those in the control group (75% and 47%, control group vs. LEAF group; 79% and 62%, control group vs. axitinib group) and the proportion and number of TDECs increased (25% and 53%, control group vs. LEAF group; 21% and 38%, control group vs. axitinib group) (Figure 2N).

The administration of axitinib to the 005 mouse model had no prognostic survival effect, but on the contrary induced tumor vascular growth.

### 3.3 Differences in Ex Vivo Angiogenesis and Genetic Characteristics Between TDECs and Normal Vascular Endothelial Cells

**FIGURE 3:**
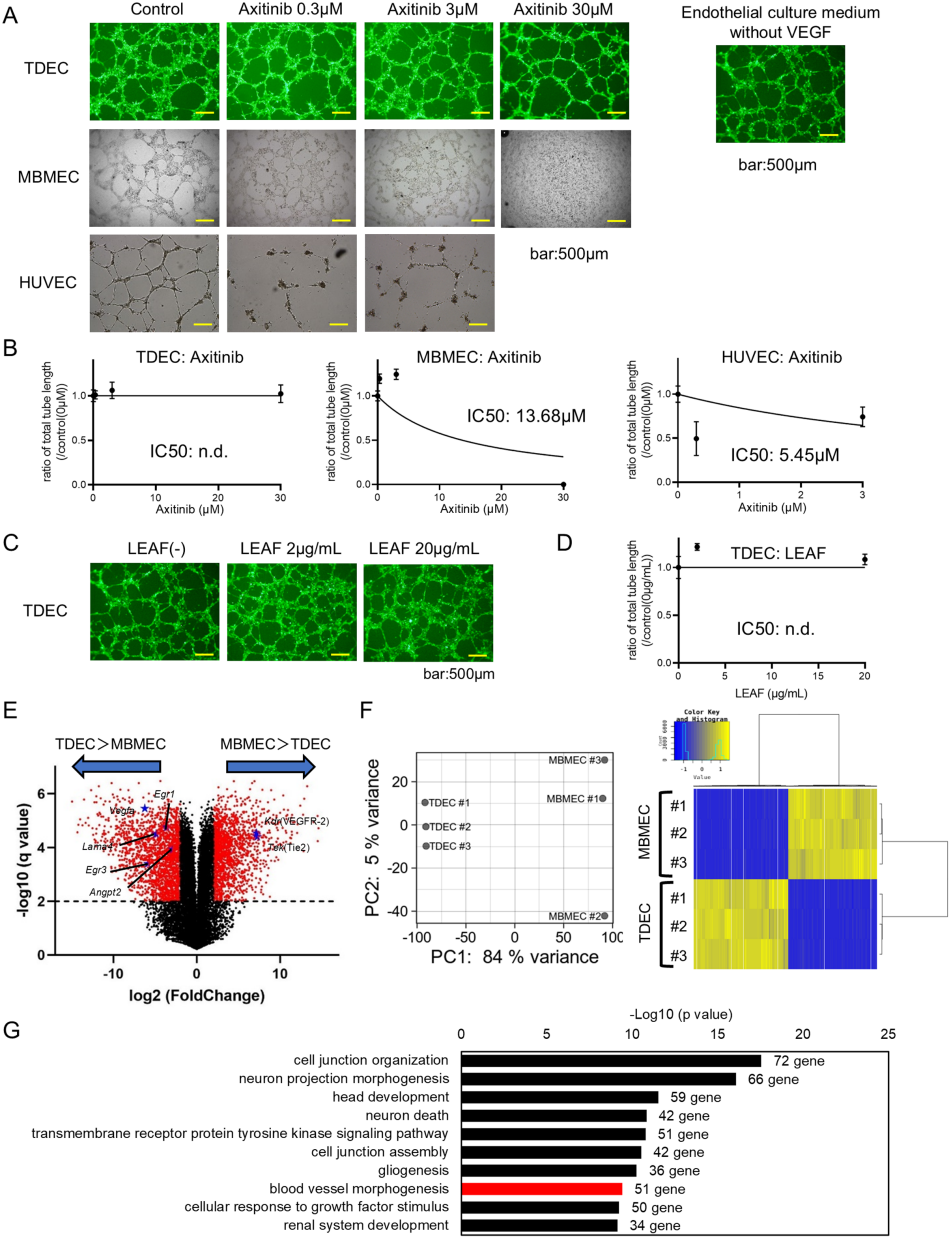
Response of TDECs and mouse brain microvascular endothelial cells (MBMECs) to anti‐angiogenic drugs and genetic analysis. (A) Tube formation assay using vascular endothelial cells at 24 h and evaluation of anti‐angiogenic effects under axitinib administration or VEGF‐free conditions (bar: 500 μm). (B) Quantitative results of total tube length of tube formation of TDECs, MBMECs, and HUVECs treated with axitinib. (*n* = 4). (C) Tube formation assay using TDECs with LEAF (bar: 500 μm). (D) Quantitative results of total tube length of TDECs treated with LEAF (*n* = 4). (E) Volcano plot analysis of TDECs and MBMECs (*q* < 0.05, log_2_ fold change (gene expression of MBMEC/TDEC) > 1 or < −1). F, Principal component analysis (PCA) and heatmap of all expressed genes of MBMECs versus TDECs detected by RNA‐Seq (*n* = 3). (G) Enrichment analysis of GO terms associated with up‐regulated genes in TDECs compared with genes in MBMECs (*q* < 0.05, log_2_ fold change (gene expression of TDEC/MBMEC) > 1).

The tube formation assays were performed to evaluate the anti‐angiogenic effects ex vivo on 3 types of endothelial cells (TDECs, MBMECs and HUVECs). Axitinib administration disrupted the tube formations of MBMECs and HUVECs in a concentration‐dependent manner; the half maximal inhibitory concentrations (IC50) were 13.68 μM (MBMEC) and 5.45 μM (HUVEC) (Figure 3A,B). In contrast, axitinib and LEAF had no impact on TDEC tube formation (Figure 3A,C,D). VEGFR‐2 was not expressed in TDECs (Figure 1H). These results suggested that tube formation of TDECs occurred through an VEGF‐independent angiogenesis pathway. On the other hand, undifferentiated 005 and U87ΔEGFR did not show obvious tube formation in culture at 24 h on Matrigel (Figure S3). Volcano plots from RNA‐seq data of MBMECs and TDECs showed that Kdr (VEGFR‐2) and Tek (Tie2) were significantly decreased in the TDECs compared with those in the MBMECs (Figure 3E). We analyzed the significantly upregulated genes in TDECs related to tumor growth factors and selected Angpt2, Lama4, Egr1, Egr3, and Vegfa. Principal component analysis and hierarchical clustering heatmap and enrichment analysis revealed that the TDEC group was enriched in blood vessel morphogenesis (51 genes, −LogP = 9.416) compared with the MBMEC group (Figure 3F,G and Figure S2). Gene signature of TDEC group highly associated with vascular proliferation.

### 3.4 Screening of New Anti‐Angiogenic Agents Targeting TDEC Tumor Blood Vessels by Drug Repositioning

**FIGURE 4:**
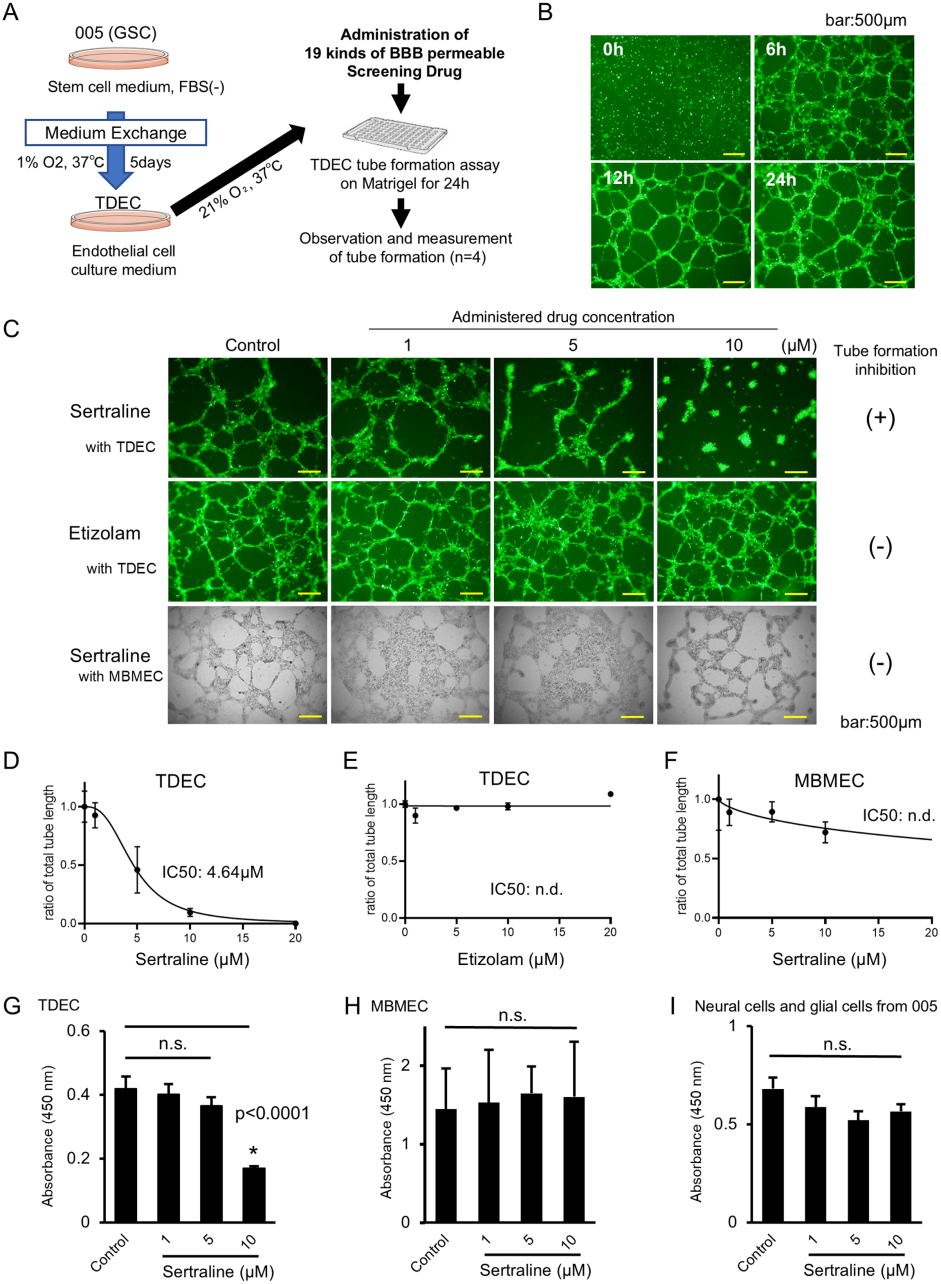
Drug screening using tube formation assays. (A) The Schema of drug screening using the ex vivo TDEC tube formation assay. (B) Fluorescence imaging of tube formation on Matrigel using TDECs differentiated from 005 cells (bar: 500 μm). (C) Tube formation assays in TDECs or MBMECs treated with the candidate drugs (bar: 500 μm). (D–F) IC50 of the inhibitory effect of the candidate drugs for TDEC‐ or MBMEC‐derived angiogenesis. (G–I) Cytotoxicity of sertraline for TDECs (G), MBMECs (H), or neural cells and glial cells from 005 (I) was measured using WST‐1 assays. * indicates significant difference.

Based on the above results and previous reports, we used the TDEC tube formation assay as an ex vivo VEGF‐independent tumor vascular screening model [23, 24, 25]. Total tube length was measured and its IC50 was evaluated to quantitatively compare screening drugs and to determine the drug concentration to be used in the mouse model. Furthermore, when targeting glioblastoma that highly infiltrates the normal brain, novel candidate drugs must pass through the BBB. We listed 19 antidepressants and anxiolytics with demonstrated ability to cross the BBB and with relatively low toxicity (Figure 4A and Figure S4). Tube formation of TDECs was detected at 6 h and was almost completed in 24 h (Figure 4B). The results showed that sertraline, a selective serotonin reuptake inhibitor (SSRI) antidepressant, had the strongest concentration‐dependent inhibitory effect on tube formation of TDECs, while etizolam, a benzodiazepine anxiolytic, had no inhibitory effect (Figure 4C–E). We further found that sertraline had no inhibitory effect on tube formation of MBMECs (Figure 4C,F). The IC50 of sertraline for tube formation of TDECs was 4.64 μM, which was the lowest level of the 19 drugs (Figure 4D; Figures S5 and S6). We next examined the growth inhibitory activity of sertraline on two types of endothelial cells (TDECs and MBMECs) and neural cells and glial cells differentiated from 005 by WST‐1 assays. Sertraline showed a growth inhibitory effect against TDECs at 10 μM, whereas no growth inhibition was observed in the other two groups up to 10 μM (Figure 4G–I and Figure S7).

These results suggest that sertraline, identified in an ex vivo drug screening, may be a potential and safe candidate as a novel anti‐angiogenic drug targeting TDECs.

### 3.5 Anti‐Angiogenic Mechanism of Sertraline in Tumor Blood Vessels Composed of TDECs

**FIGURE 5:**
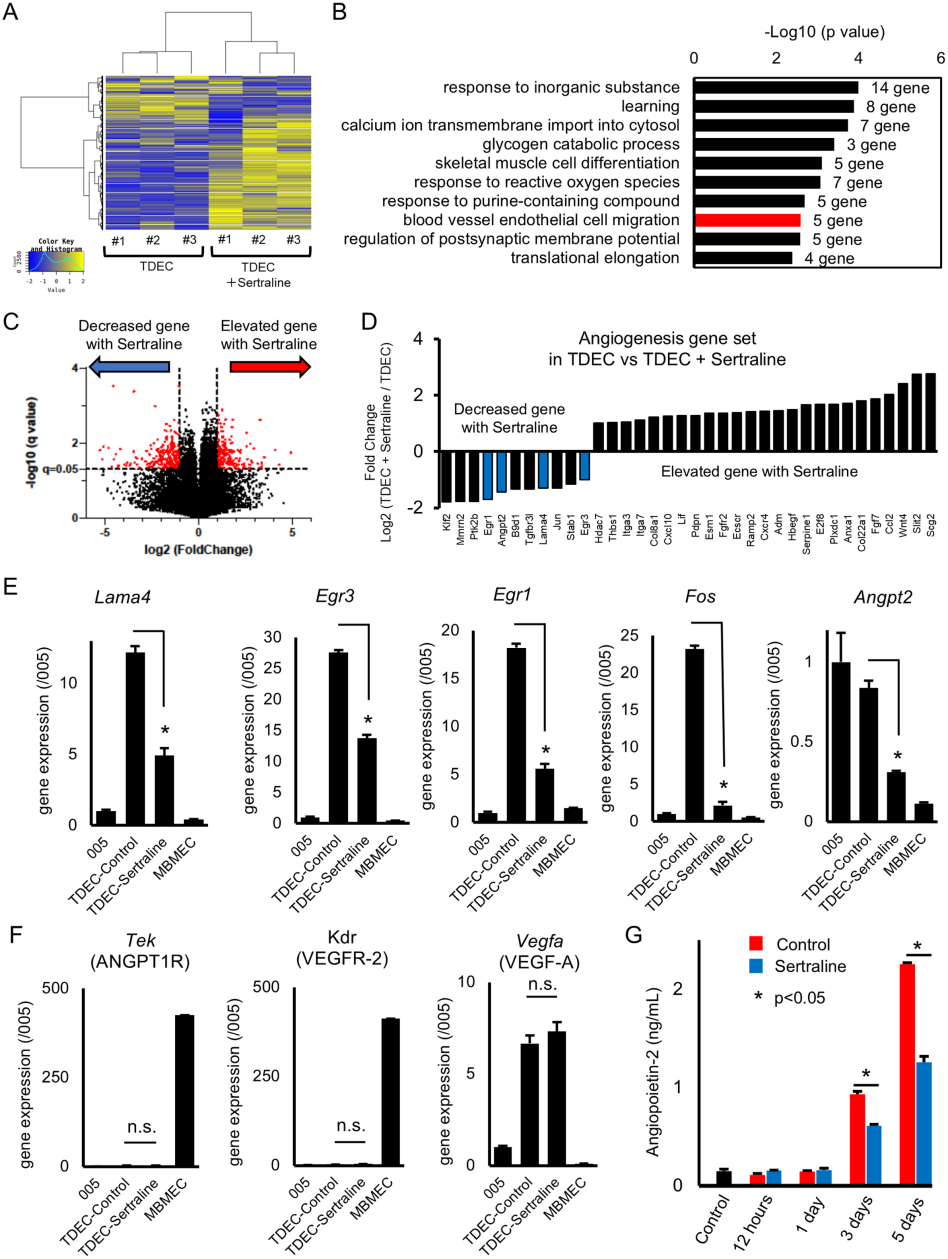
RNA‐seq of TDECs treated with sertraline. (A) Hierarchical clustering heatmap using RNA‐seq in control TDECs and TDECs treated with sertraline for 24 h (each *n* = 3). (B) Enrichment analysis of GO terms associated with down‐regulated genes in treated TDECs compared with genes in control TDECs (*q* < 0.05, log_2_ fold change (gene expression of treated/controls < −1). (C) Volcano plot analysis of data from RNA‐seq (*q* < 0.05, log_2_ fold change (gene expression of treated/controls) > 1 or < −1). D, Angiogenesis gene set expressions in treated TDECs compared with those in control TDECs (log_2_ fold change (gene expression of treated/controls) > 1 or < −1). (E, F) Gene expression of genes associated with vascular proliferation in 005 cells, TDECs, TDECs treated with sertraline, and MBMECs (Lama4: *p* = 0.0001, Egr3: *p* = 0.0027, Egr1: *p* < 0.0001, Fos: *p* < 0.0001, Angpt2: *p* < 0.0001, and Tek, Kdr, and Vegfa: *p* > 0.05, TDECs treated with sertraline vs. TDECs). (G) ELISA of Angiopoieting‐2 secreted into TDEC culture medium. * indicates significant difference.

We performed in vitro RNA‐seq on TDECs before and after sertraline (5 μM) administration (biological replicates, *n* = 3) and hierarchical clustering heatmap (Figure 5A). We focused on the gene signature related to blood vessel endothelial cell migration in enrichment analysis (−LogP = 2.590) (Figure 5B and Figure S1). We also focused on angiogenesis‐related genes that were significantly suppressed after sertraline administration from genes differentiated by the volcano plot (*q* = 0.05, Log_2_FC < −1 or 1 < Log_2_FC) (Figure 5C,D) and selected 5 genes, Lama4, Egr3, Egr1, Fos, and Angpt2. Egr3, Egr1, and Fos are VEGF pathway‐dependent vascular growth factors, and Lama4 and Angpt2 are VEGF‐independent vascular growth factors. We examined the mRNA expression of the 5 genes in 005 cells and MBMECs (Figure 5E). Kdr (VEGFR‐2) and Vegfa (VEGFA) genes, which play an important role in the VEGF pathway, were also evaluated, but these were not affected by sertraline administration. In addition, the expression of Tek (ANGPT1R, a receptor for Angiopoietin‐2), was low in 3 groups except for MBMECs (Figure 5F). There were no expressions of the sertraline target receptor, SLC6A4 (Serotonin Transporter), in 005 cells and TDECs. In the secreted Angiopoietin‐2 treated with sertraline, the increase of Angiopoietin‐2 in controls on Days 3 and 5 and a suppression of Angiopoietin‐2 secretion in the sertraline‐administered group at both time points were observed (Figure 5G). These results showed that sertraline had a broad spectrum of anti‐angiogenic effects, mainly on the non‐VEGF pathway.

### 3.6 Combination of Axitinib and Sertraline Prolonged Survival in a GSC Mouse Brain Tumor Model Resistant to VEGF Pathway Inhibitors

**FIGURE 6:**
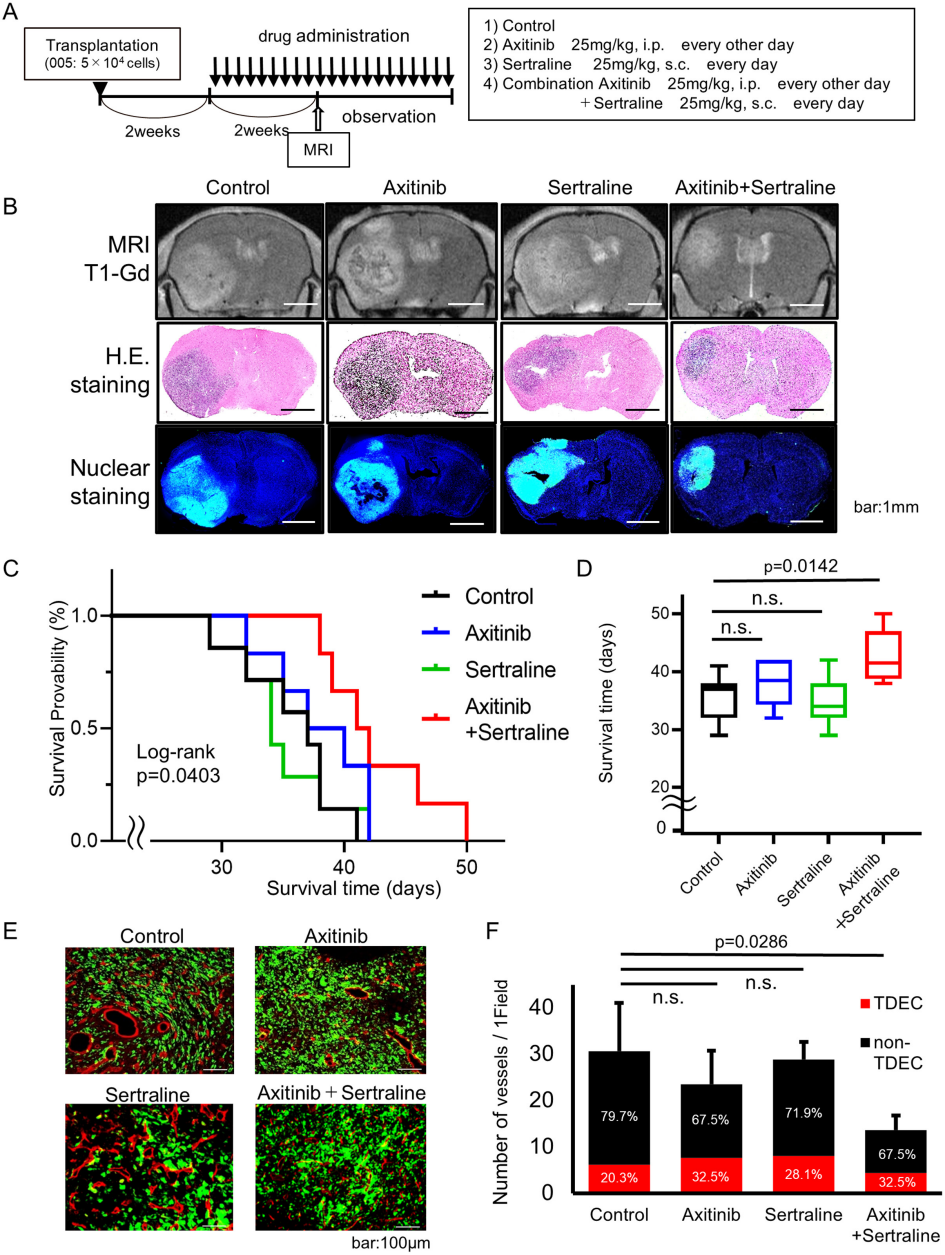
Combination treatment of axitinib and sertraline in 005 mouse model. (A) Experimental overview. (B) T1‐Gadolinium (Gd) image of MRI and HE‐stained and nuclear‐stained histology in tumor sections 2 weeks after treatment (scale bar: 1 mm). (C) Kaplan–Meier survival curves of the indicated groups (each *n* = 6 or 7) (log‐rank test *p* = 0.0403). (D) Graph of median survival time of the indicated groups (each *n* = 6 or 7) (control vs. combination of axitinib and sertraline, unpaired *t*‐test, *p* = 0.0142). (E) Comparison of tumor blood vessels in tumors by IHC of CD31 in the indicated groups. (F) Number of tumor blood vessels in tumors in the indicated groups and the ratio of TDEC/non‐TDEC in IHC data (control vs. combination of axitinib and sertraline: *p* = 0.0286).

At 2 weeks after intracerebral transplantation of 005 cells, mice were treated with sertraline alone or combined with axitinib (Figure 6A–D). The axitinib monotherapy had no therapeutic effect in 005 mouse model (Figures 2H and 6C,D). MRI and IHC of the tumors at 4 weeks after transplantation and 2 weeks after treatment showed a trend toward tumor reduction in the axitinib + sertraline combination group compared with the control, axitinib, and sertraline groups (Figure 6B). Kaplan–Meier survival curve analysis showed improved survival days in the axitinib + sertraline combination group (log‐rank test *p* = 0.0403) (Figure 6C). There were no differences between the median survival days of the control, axitinib and sertraline groups (35.7 ± 1.42 (SE), 38.0 ± 1.51 (SE) and 34.9 ± 1.5 (SE) days, respectively); however, the median survival days was 42.7 ± 1.69 (SE) days in the axitinib + sertraline combination group, indicating a prognostic benefit of approximately 20% (unpaired *t*‐test, *p* = 0.0142) (Figure 6D). In brain tumor samples on Day 28, tumor blood vessels of both types (TDECs and non‐TDECs) were significantly reduced in the axitinib + sertraline combination group compared with those in the control group (*p* = 0.0286) (Figure 6E,F). These results in vivo suggest that sertraline alone has no effect on the differentiation or growth of GSCs. Furthermore, in the combination group, some necrosis from ischemia was observed as well as marked tumor vascular inhibition (Figure 6E,F).

**FIGURE 7:**
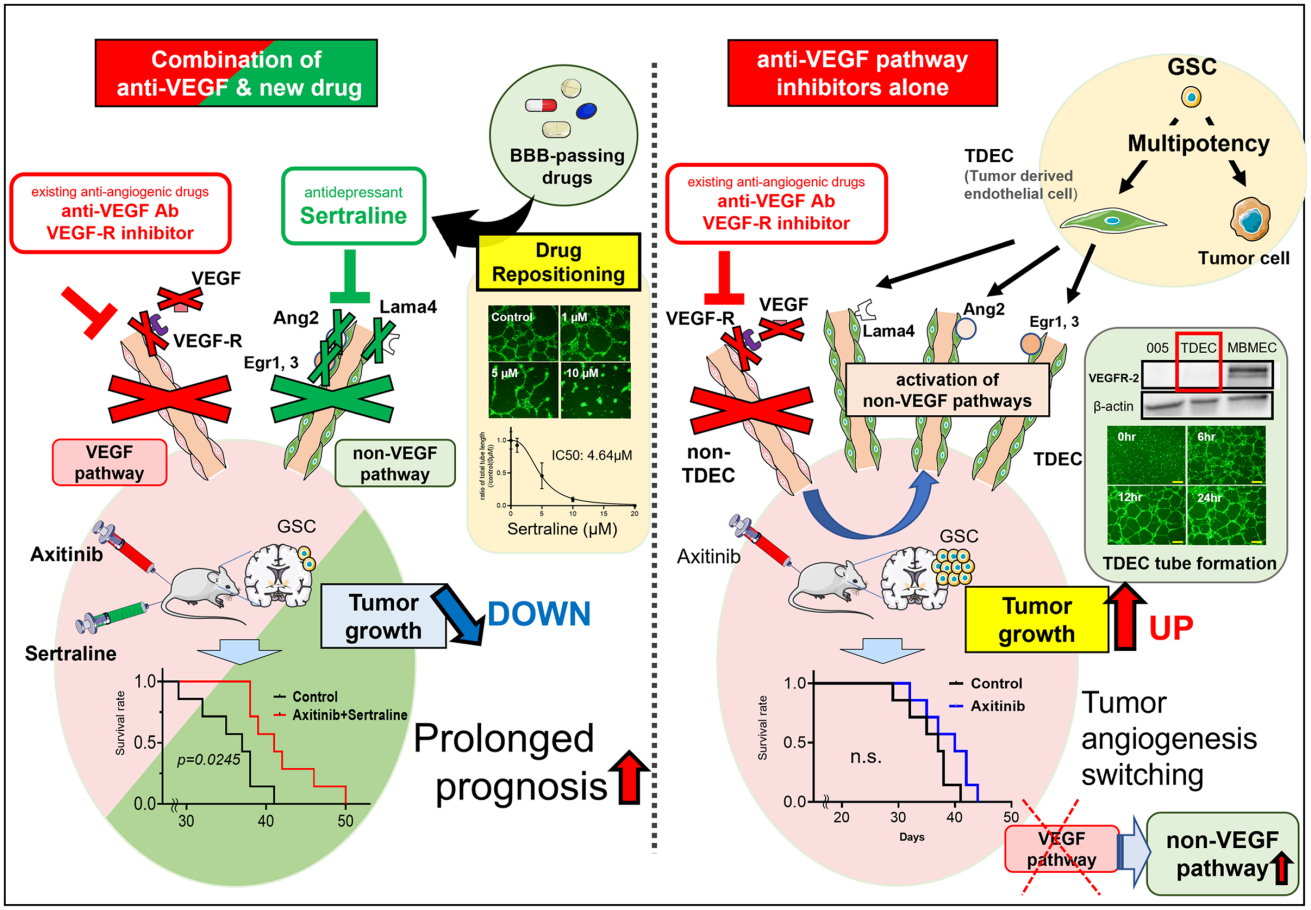
Graphic abstract of combined sertraline and anti‐VEGF therapy to glioblastoma. Right: Anti‐angiogenic therapy with VEGF pathway inhibition induces TDEC differentiation from glioma stem like cells, resulting in no therapeutic effect. Left: Combination therapy with a VEGF pathway inhibitor and sertraline (SSRI), TDEC‐targeted drug discovered through drug repositioning screening, provides new anti‐tumor and prognostic effects.

These data indicated that one of the causes of resistance to anti‐VEGF pathway inhibitors was increased angiogenesis by TDECs differentiated from GSCs. They also indicated that the combination of anti‐VEGF pathway inhibitors and sertraline prolonged survival (Figure 7).

## 4 Discussion

The key points of this study are “focusing on tumor blood vessels consisting of TDECs differentiated from GSCs as one of the causes of resistance to anti‐VEGF pathway inhibitors” and “developing novel angiogenesis inhibitors for TDECs by repositioning existing drugs in the BBB passage”. Anti‐VEGF pathway inhibitors are an attractive therapy for various cancers but have limited therapeutic effects for glioblastoma, such as improvement of radiation necrosis and improvement of ADL because of reduced edema [26, 27, 28]. Fluvoxamine, an antidepressant identified by drug repositioning, shows efficacy against glioblastoma tumor invasion through the inhibition of invasive process formation [29]. Another study showed that the combination of the antidepressant imipramine and bevacizumab is effective in immunopotentiation [30]. Targeting glioblastoma, a highly invasive cancer, requires strategic therapy, such as combining BBB‐passing agents with other anticancer agents [30]. In this study, we demonstrated the efficacy of a combination therapy strategy using antiangiogenic therapy using anti‐VEGF pathway inhibitors. Targeting the tumor vasculature is a potent therapeutic strategy for glioblastoma as well as other malignancies.

The tumor vasculature in glioblastoma is composed of various types of endothelial cells (ECs), including normal brain ECs, bone marrow‐derived ECs, mesenchymal stem cell–derived ECs, and TDECs [18]. In this study, we focused on the effects of tumor vessels composed of TDECs differentiated from GSCs and developed therapeutic agents targeting TDECs tumor vessels. Previous reports and our results suggested that some GSCs and TDECs do not express VEGFR‐2 and exhibit angiogenic activity through a non‐VEGF pathway [31]. Although TDECs were relatively rare in untreated tumors, we found that anti‐VEGF pathway inhibition significantly increased TDECs in a reactive manner [32]. Thus, anti‐angiogenic therapies that inhibit the VEGF pathway induced endothelial cell switching in tumor vessels, one of the causes of treatment resistance. Targeting TDECs complements the limitations of anti‐VEGF antibody therapy, with reduced side effects on normal blood vessels. Various clinical trials have been conducted on treatments in primary and recurrent glioblastoma that target the tumor vasculature through non‐VEGF pathway factors [33, 34]. These factors include the Angiopoietin‐2 (PF‐04856884), PDGFR (platelet‐derived growth factor receptor) and FLT3 (FMS‐like tyrosine kinase 3) (MLN518), EGF, HER2 and Ephrin B4 (NCT02844439). However, the clinical trials have not yielded the expected results [35, 36, 37, 38]. Novel anti‐angiogenic inhibitors for non‐VEGF pathways in other malignancies have been explored in glioblastoma, but the BBB is a problem for drug delivery [39]. The advantages of the drug positioning approach are that the candidate drugs are already available and have been demonstrated as safe, which significantly reduces drug development costs. Notably, we focused on drugs that reliably pass the BBB, which is the biggest obstacle to developing therapeutic agents for glioblastoma. Additionally, antidepressants are known to accumulate in high concentrations in the brain [40].

Some reports suggest that antidepressant use in glioblastoma does not prolong overall survival (OS) [41]. However, the Mayo Clinic reported a trend toward improved prognosis in patients with glioblastoma in the group that used antidepressants in addition to standard therapy [42]. In addition, the anti‐tumor effect of SSRI was observed at the animal experimental level [29]. Our results showed no expression of SLC6A4 (Serotonin Transporter), the target receptor for sertraline. Sertraline was thought to act through a pathway different from the serotonin pathway similar to other SSRIs. Our gene analysis results suggest that the anti‐angiogenic mechanism of sertraline may occur through the inhibition of TDEC‐derived angiogenesis via suppression of various pathways and genes including Angpt2, Lama4, Egr1, Egr3, and Fos genes. Angiopoietin‐2 (Angpt2) acts as both an agonist and antagonist for the TIE2 receptor. The conditions depend on the concentration of angiopoietin‐2 and the environment of the secretory cell, such as inflammation, tumor, and the presence of integrins [43, 44]. LAMA‐4, Laminin Subunit Alpha 4, is specifically secreted by brain tumor cell lines and gliomas. One study showed that a higher patient grade is associated with a greater amount of LAMA‐4 secreted into the cerebrospinal fluid [45].

LAMA‐4 inhibits tumor cell migration and growth along blood vessels and is thus an important target for tumor cell invasion and angiogenesis [46]. In the present study, we found that LAMA‐4 expression increased during the differentiation of GSCs into TDECs and that its expression was suppressed by sertraline. LAMA‐4 is not expressed in normal vascular endothelial cells; both Angiopietin‐2 and LAMA‐4 are expressed in renal cell carcinoma and have been reported as prognostic factors [47, 48]. The combination of an Ang/Tie‐2 inhibitor (trebananib) targeting Ang‐1, Ang‐2, and Tie‐2 receptors with a VEGF inhibitor was expected to have an effective anti‐tumor effect on glioblastoma but did not show a prognostic benefit in a clinical trial (NCT01609790) [49]. Upregulation of compensatory mechanisms by VEGF inhibitors is difficult to overcome with a single pathway inhibition combination. Multiple pathway inhibition, including Angiopoietin‐2 and LAMA‐4, could be meaningful due to changes in multiple genes, as in the present study with Sertraline, and further detailed exploration of pharmacological mechanisms is required.

In conclusion, our results suggested that TDECs may be one of the causes of resistance to anti‐VEGF pathway inhibitors in the 005. These results suggested that sertraline was one of the novel therapeutic candidates against TDECs and could safely and effectively improve the prognosis of glioblastoma patients. Anti‐angiogenic treatment combinations targeting various pathways showed the potential to be a new treatment option for glioblastoma.

## Author Contributions

**Nobushige Tsuboi:** data curation (lead), formal analysis (lead), methodology (lead), project administration (equal), resources (lead), software (lead), validation (lead), visualization (lead), writing – original draft (equal). **Yoshihiro Otani:** methodology (equal), resources (supporting), supervision (equal). **Atsuhito Uneda:** formal analysis (supporting), methodology (supporting), software (supporting), supervision (equal). **Joji Ishida:** investigation (supporting), supervision (equal). **Yasuki Suruga:** project administration (equal). **Yuji Matsumoto:** supervision (equal). **Atsushi Fujimura:** methodology (supporting), supervision (equal). **Kentaro Fujii:** supervision (equal). **Hideki Matsui:** investigation (supporting), resources (supporting). **Kazuhiko Kurozumi:** investigation (supporting), supervision (equal). **Isao Date:** investigation (supporting), resources (equal), supervision (equal). **Hiroyuki Michiue:** conceptualization (lead), funding acquisition (lead), investigation (equal), project administration (lead), resources (equal), supervision (equal), visualization (equal), writing – original draft (equal), writing – review and editing (lead).

## Ethics Statement

This study was approved by the research ethics committee of the Okayama University (approval nos. G1608‐026 and 1911–023). All animal studies were performed following Okayama University ethical guidelines for experimental animal care (OKU‐2018838, OKU‐2019568, OKU‐2020793, OKU‐2021590).

## Conflicts of Interest

The authors declare no conflicts of interest.

## Supporting information


Data S1.

## Data Availability

The article includes all data in the study, and further inquiries can be directed to the corresponding author.
