# Supplementary material for: New Anti‐Angiogenic Therapy for Glioblastoma With the Anti‐Depressant Sertraline
Source: Cancer Med. 2024 Oct 23;13(20):e70288. doi: 10.1002/cam4.70288 (PMC11497491; doi:10.1002/cam4.70288)

List of Supporting Information

Supplementary Figure 1

Differentiation experiments of GSCs 005 and U87ΔEGFR into TDEC under different culture conditions

Supplementary Figure 2

GO terms and each respective genes in enrichment analysis

Supplementary Figure 3

Microscope imaging of tube formation assay of non-differentiated 005 cells and U87ΔEGFR for 24 h

Supplementary Figure 4

List of 19 candidate drugs that pass through the blood-brain barrier for drug repositioning in this paper.

Supplementary Figure 5

Fluorescence microscope image of TDECs tube formation assay with 19 different drugs at various concentrations for 24 hours

Supplementary Figure 6

Quantitative evaluation of the inhibitory effect on tube formation was presented in Supplementary Figure 5.

Supplementary Figure 7

Evaluation of cytotoxicity of drugs to TDECs

Supplementary Figures

Supplementary Figure 1

Differentiation experiments of GSCs 005 and U87ΔEGFR into TDEC under different culture conditions

A. Immunocytochemistry of 005 cells cultured in DMEM and 10%FBS, same as the cells in Figure. 1D, stained for CD31 and CD34. (bar: 10μm)

B. Immunocytochemistry of U87 Δ EGFR cells cultured under the same condition that is for TDEC differentiation stained for HLA, CD31, and CD34. (bar: 20μm)

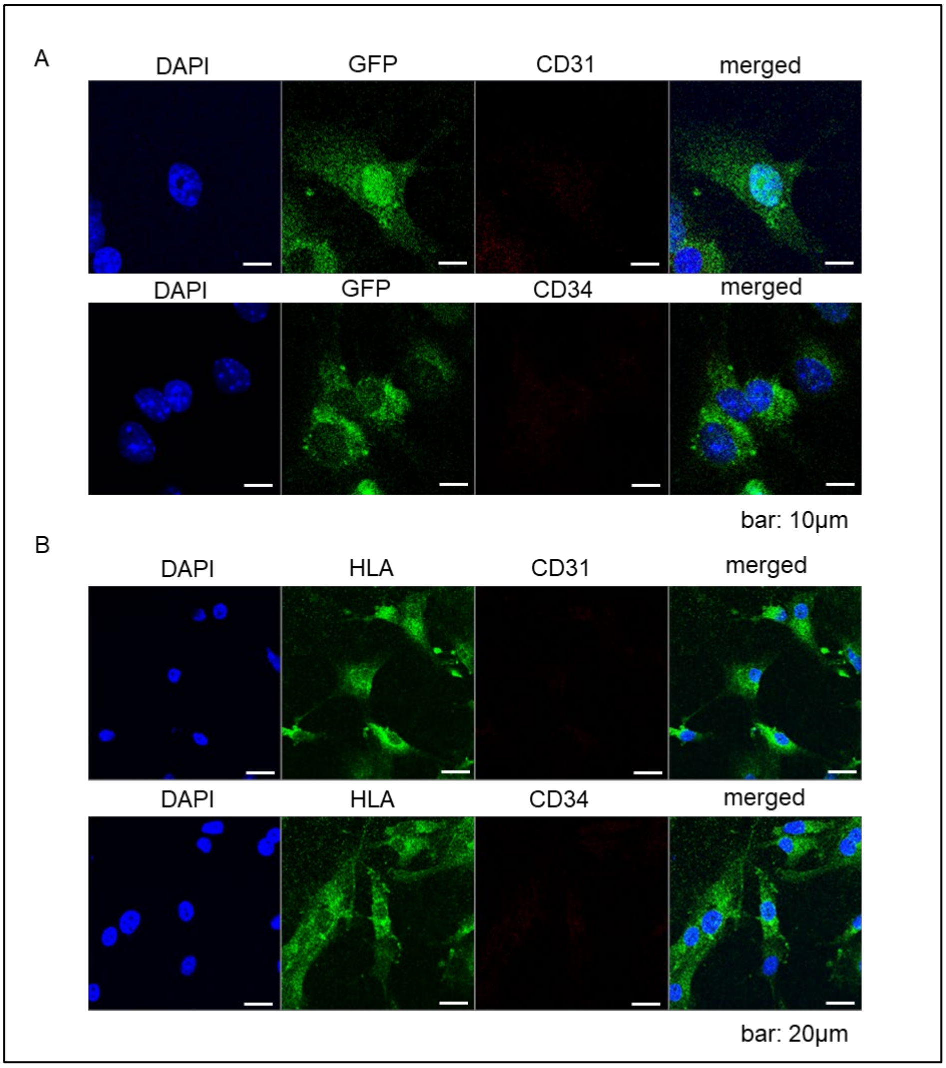

Supplementary Figure 2

GO terms and each respective genes in enrichment analysis

GO terms and gene symbols of the genes picked up by enrichment analysis in Fig1G, 3G, and 5B.

	Description	GO Term	Symbols
Fig. 1G	blood vessel morphogenesis	GO:0048514	Ace,Adm,Agt,Apoe,Rhob,Bsg,C3,Anxa2,Cdh13,Col18a1,Co<br>l8a1,Gadd45a,Ecm1,Efnb2,Egf,Egr3,Emp2,Epas1,Epha1,E<br>pha2,Ephb2,F3,Fgfbp1,Ccn2,Fkbp10,Flt1,Fn1,Fut1,Glul,Lrp<br>2,Grn,Hbegf,Hk2,Hmga2,Hmox1,Nr4a1,Hpse,Hyal1,Id1,Cc<br>n1,Itga7,Itgb2,Jag1,Junb,Jup,Kdr,Klf2,Klf4,Lama1,Lif,Lrp1,S<br>mad6,Smad7,Mdk,Foxc1,Myh9,Notch2,Pdgfb,Pdgfrb,Pgk1,<br>Prkcb,Plcd1,Serpinf2,Ptgs2,Ptk2b,Rora,Sars,Scg2,Cx3cl1,S<br>erpinf1,Sema3e,Sgpl1,Sphk1,Stra6,Abcc8,Tbx2,Tgfb1,Thbs<br>1,Tnfaip2,Vegfa,Wars,Wnt5a,Xbp1,Xdh,Fbln5,Ceacam1,Dy<br>sf,Tnfrsf12a,Hif3a,Ramp3,Mmp19,Ndnf,Angptl6,Myo1e,Plxd<br>c1,Lrg1,Loxl2,Tiparp,Mmrn2,Card10,Unc5b,Adgrb1,Cela1,<br>Egln1,Stab1,Flcn,Adm2,Ppp1r16b,Jcad,Ism1,Atp2b4,Tgfbr3<br>l,Ddit3,Egr1,Il6ra,Lama4,Nfe2,Wnt2,Llgl2,Robo2,Ltbp1,Rom<br>1,Errfi1,Cth,Adora2b,Adra2a,Ager,Aldoa,Aoc3,Rhoc,Bmp2,<br>Calr,Cpeb1,Csf1,Dab2,Dmtn,Fpr2,Gata3,Gcnt2,Gpi1,Hspa5<br>,Igf1r,Igfbp5,Il16,Il1r1,Il6st,Itga2b,Itga3,Lamb1,Smad3,Met,<br>Myo1f,Nedd9,P2rx4,P2ry2,Pld2,Cavin1,Reln,Selp,Sema3b,<br>Stat5a,Swap70,Trf,Vil1,Wnt5b,Tlr2,Pla2g7,Dock5,Atoh8,Il3<br>4,Slc26a5,Akap12,Il23a,Sucnr1,Gpnmb,Fermt3,Synpo2,Se<br>ma3g,Plcg2,Fam107a,Cass4,Lgr6,Areg,Cdkn2a,Cdkn2b,C<br>ebpb,Ctsl,Dlk1,Ednrb,Hpn,Igfbp3,Igfbp4,Klf9,Nme2,Pax2,Pt<br>prn,Stat6,Tfap2c,Tlr4,Vdr,Zfp36,Sfn,Nupr1,Ift172,Wfdc1,Zfa<br>s1,Pold4,Ern1,Foxp1,Esrp1,Mcc,Trim46,Stc1,Dnaja4,Lama<br>5,Sdc4,Arhgap4,Itgb4,Appl2,Radil
Fig. 3G	blood vessel morphogenesis	GO:0048514	Agt,Apoe,Prdm1,Bmpr1a,Emp2,Epn2,F3,Fgfr1,Gata6,Has2,<br>Hey1,Hk2,Hmox1,Jun,Lama1,Lrp1,Ppp1r15a,Notch2,Notch<br>3,Nrxn1,Ntrk2,Prrx1,Ptn,Rbpj,Sars,Scg2,Sema5a,Sgpl1,Sta<br>t3,Tcf4,Ubp1,Vav2,Vegfa,Wnt7b,Spry2,Efemp2,Rgcc,Ddah<br>1,Angptl6,Emilin1,Adamts9,Tafa5,Unc5b,Jmjd6,Adgrb1,Egl<br>n1,Spred1,Adgrb2,Tspan12,Ephb1,Zmiz1,Dhcr7,Egr1,Ptk7,<br>Cth,Slc4a7
Fig. 5B	blood vessel endothelial cell migration	GO:0043534	Angpt2,Egr3,Fgfbp1,Ptk2b,Mmrn2,Egr1,Fos,Hes5,Ier2,Jun,<br>Ptprk,Scx,Zfp36,Wfikkn1,Tgfbr3l,Klf2

Supplementary Figure 3

Microscope imaging of tube formation assay of non-differentiated 005 cells and U87ΔEGFR for 24 hr

A: 005 with GFP and bright field (3x10^4 cells /1well)

B: U87ΔEGFR with bright field (2x10^4 cells or 3x10^4cells /1well) (bar: 1mm)

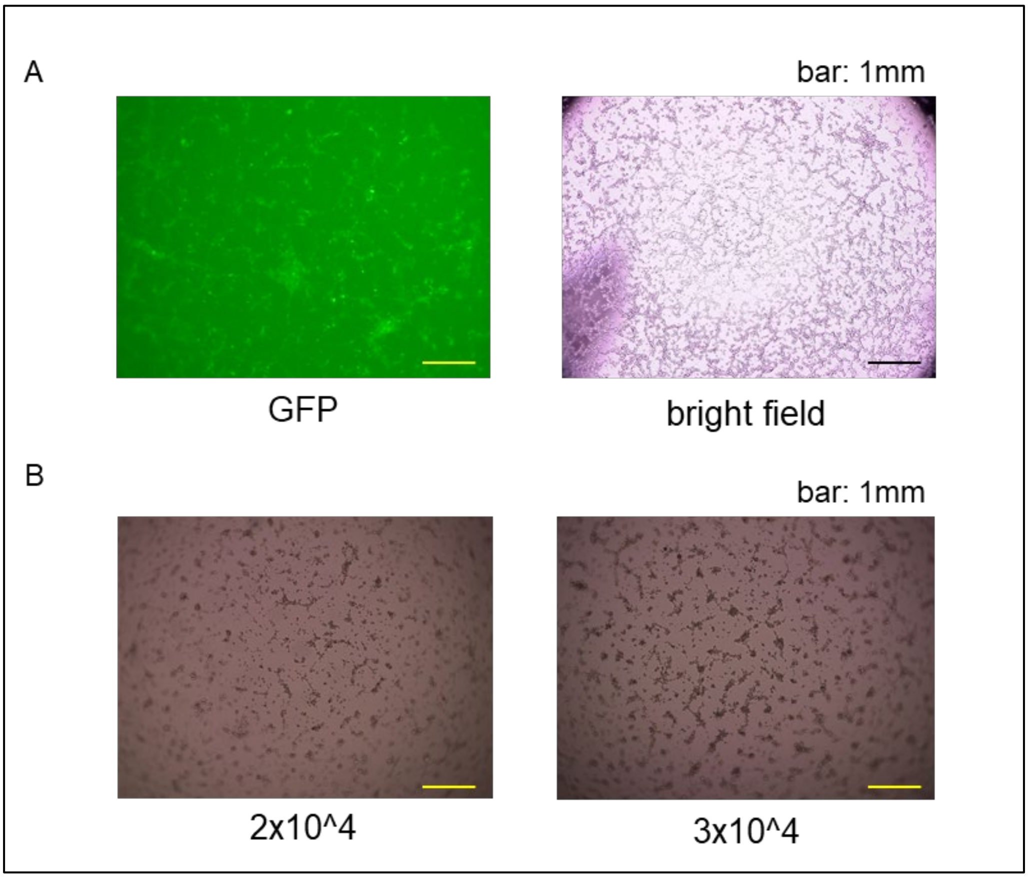

Supplementary Figure 4

List of 19 candidate drugs that pass through the blood-brain barrier for drug repositioning in this paper.

Type	Drug name	Manufacturer	IC50(μM)
Typical antipsychotic	Chlorpromazine	Tokyo Chemical Industry Co., Ltd.	4.863
Atypical antipsychotic	Olanzapine	Tokyo Chemical Industry Co., Ltd.	n.d.
	Quetiapine	FUJIFILM Wako Pure Chemical Corporation	6.964
Benzodiazepine	Etizolam	FUJIFILM Wako Pure Chemical Corporation	n.d.
GABA-R antagonist	Flumazenil	Tokyo Chemical Industry Co., Ltd.	n.d.
Tricyclic antidepressant	Imipramine	Tokyo Chemical Industry Co., Ltd.	118.2
	Clomipramine	Cayman Chemical Company	32.13
	Lofepramine	Cayman Chemical Company	n.d.
	Trimipramine	Toronto Research Chemicals Inc,	116.7
	Amitriptyline	Cayman Chemical Company	57.08
Tetracyclic antidepressant	Mianserin	Tokyo Chemical Industry Co., Ltd.	24.37
SSRI	Paroxetine	Tokyo Chemical Industry Co., Ltd.	13.43
	Fluoxetine	Tokyo Chemical Industry Co., Ltd.	6.161
	Fluvoxamine	Tokyo Chemical Industry Co., Ltd.	n.d.
	Sertraline	Tokyo Chemical Industry Co., Ltd.	4.644
	Citalopram	Tokyo Chemical Industry Co., Ltd.	133.8
SNRI	Venlafaxine	Tokyo Chemical Industry Co., Ltd.	n.d.
	Milnacipran	Tokyo Chemical Industry Co., Ltd.	86.08
	Duloxetine	Tokyo Chemical Industry Co., Ltd.	9.767

n.d., no data

Supplementary Figure 5

Fluorescence microscope image of TDECs tube formation assay with 19 different drugs at various concentrations (0, 1, 5, 10 µM) for 24 hours (bar: 1 mm).

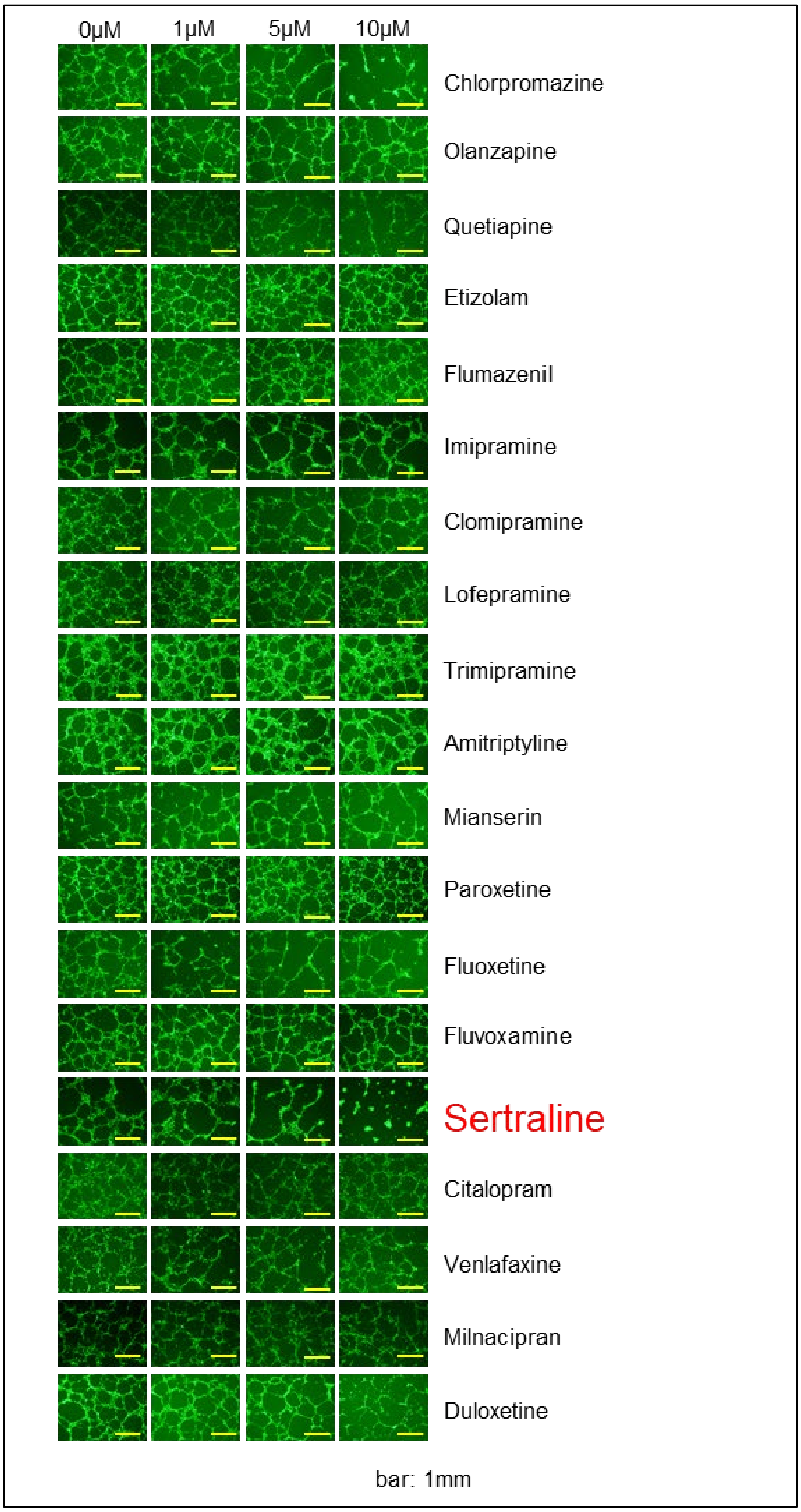

Supplementary Figure 6

Quantitative evaluation of the inhibitory effect on tube formation was presented in Supplementary Figure 5.

IC50 of each drug was calculated by measuring and graphing the total tube length of tube formation. (X-axis: concentration of each drug, Y-axis: ratio of total tube length compared to control(0μM))

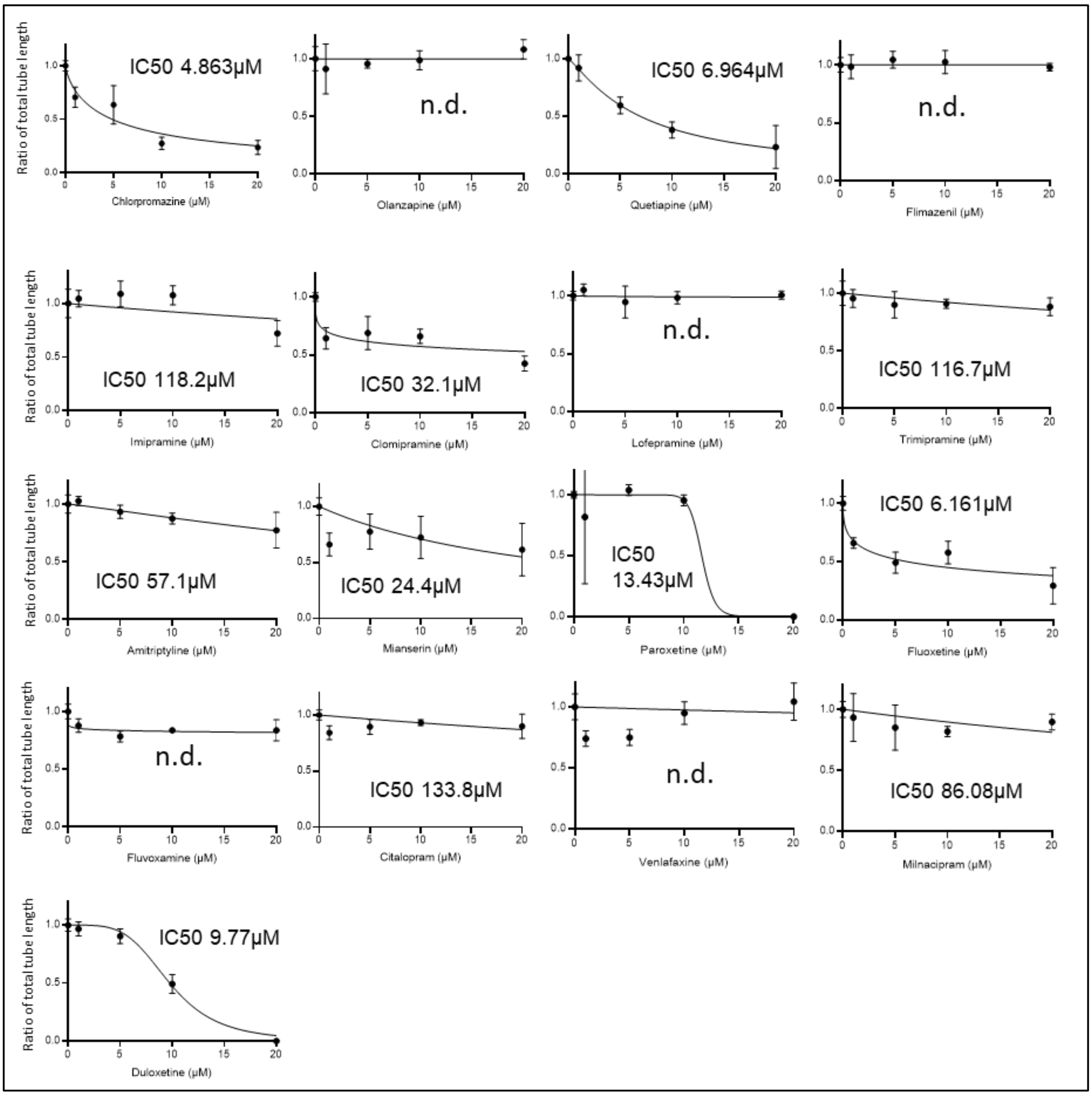

Supplementary Figure 7

Evaluation of cytotoxicity of drugs to TDECs

Evaluation of cytotoxicity of 19 drugs on TDECs. with WST-1 assay (X-axis: concentration of each drug, Y-axis: absorbance at 438nm after 4 hours of WST-1 administration)

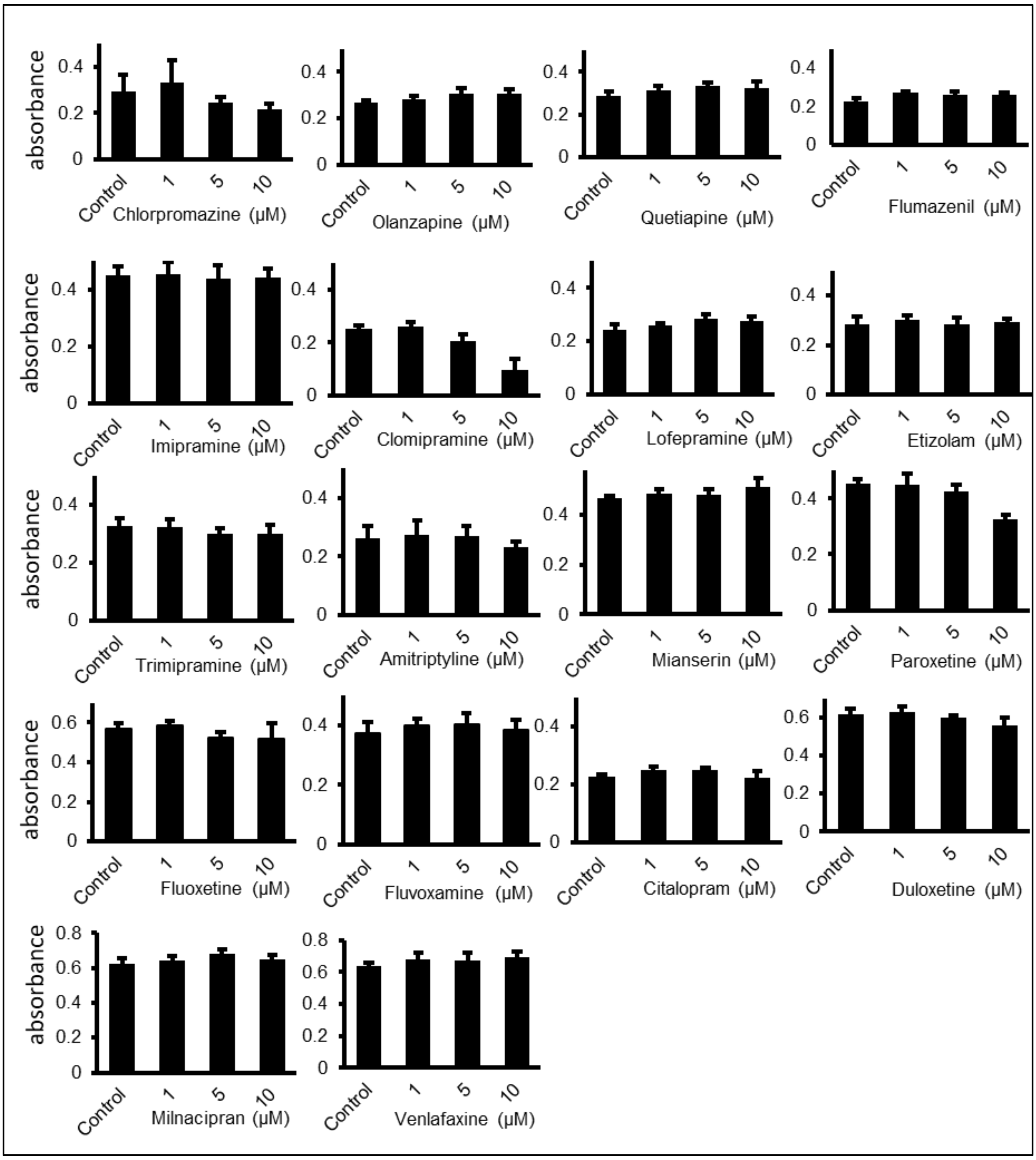

Supplement: Supplementary file 1 — Data S1. [file CAM4-13-e70288-s001.pdf]
